# Household Poverty-Wealth and Decision-Making Autonomy as Predictors of Reproductive and Maternal Health Services Utilization among Rural Women in Nigeria: Evidence from a National Survey

**DOI:** 10.4314/ejhs.v33i1.20

**Published:** 2023-01

**Authors:** Imo Chukwuechefulam Kingsley

**Affiliations:** 1 Department of Sociology, Faculty of the Social Sciences, Adekunle Ajasin University, Akoko-Akungba, Ondo State, Nigeria

**Keywords:** Reproductive, maternal health services, poverty, decision-making, rural women

## Abstract

**Background:**

Adequate reproductive and maternal healthcare services utilization are significant in reducing maternal deaths, however, the prevalence rate of contraceptive use remains low, with inadequate maternal health services utilization among rural women in Nigeria. This study examined the influence of household poverty-wealth and decision-making autonomy on reproductive and maternal health services utilization among rural women in Nigeria.

**Methods:**

The study analyzed data from a weighted sample of 13,151 currently married and cohabiting rural women. Descriptive and analytical statistics including multivariate binary logistic regression were conducted using Stata software.

**Results:**

An overwhelming majority of rural women (90.8%) have not used modern contraceptive methods, with poor utilization of maternal health services. About 25% who delivered at home received skilled postnatal checks during the first 2 days after childbirth. Household poverty-wealth significantly reduced the likelihood of using modern contraceptives (aOR: 0.66, 95% CI: 0.52–0.84), having at least four ANC visits (aOR: 0.43, 95% CI: 0.36–0.51), delivering in a health facility (aOR: 0.35, 95% CI: 0.29–0.42) and receiving a skilled postnatal check (aOR: 0.36, 95% CI: 0.15–0.88). Women's decision-making autonomy regarding their healthcare significantly increased the use of modern contraceptives and the number of ANC visits, while women's autonomy on how their earnings are spent positively influenced the use of maternal healthcare services.

**Conclusions:**

In conclusion, the use of reproductive and maternal health services among rural women was associated with household poverty-wealth and decision-making autonomy. Government should formulate more pragmatic policies that will create awareness and promote universal access to reproductive and maternal healthcare services.

## Introduction

In the context of global health priority, the levels of utilization of reproductive and maternal health services are parts of the indicators in measuring the level of healthcare performance, delivery system and developmental indices (1). The use of modern contraceptive methods, antenatal care (ANC), and postnatal care (PNC) are major key maternal health interventions that have tremendously contributed to the reduction of maternal mortality worldwide (2–3). The use of contraceptives among married childbearing women in Africa stands at 31% even with the high rate of maternal mortality (4). Sub-Saharan Africa (SSA), of which Nigeria is an integral part, has the lowest prevalence rate of contraceptive use notwithstanding the relatively high knowledge and awareness of contraception (4–5). This could account for the experience of more than 14 million unplanned pregnancies each year in the region (6) and constituted roughly 66% of the world's maternal deaths (7). The health consequences of unplanned pregnancies are remarkable public health concerns, particularly for disadvantaged women (8).

Maternal healthcare including optimal ANC visits, delivery in a health facility and skilled postnatal check after delivery are significant mediations required during pregnancy and after childbirth for positive maternal and child health outcomes (9). Although, the World Health Organization's (WHO) guidelines for the four-visit-focused ANC model recommended at least four ANC visits during pregnancy and this was associated with more maternal and perinatal deaths (10). This subsequently led to the revision of the guidelines recommending the minimum number of ANC visits from four to eight contacts throughout the pregnancy with the first visit occurring within 12 weeks (first trimester) of gestation (9). Delivering in a health facility is expected to be assisted by a skilled provider which plays a critical role in preventing stillbirths and improving newborn survival (11), but may not translate to a postnatal check from a skilled provider. However, early PNC services from skilled providers are fundamental to reducing maternal mortality rates and ending preventable deaths of newborns (12).

In sub-Saharan Africa, which Nigeria is part of, previous studies have shown the existence of urban-rural disparity in the use of maternal and reproductive health services by demonstrating that urban women tend to use modern contraceptives and maternal health services compared to rural women (13–14). Notwithstanding the progress made in achieving universal access to reproductive and maternal health services in some sub-Saharan African countries (15), substantial disparities exist between urban and rural women in Nigeria. For instance, about 18% of urban women compared to 7.8% of their rural counterparts used any modern contraceptive (16). Also, there was urban-rural disparity among women who reported having four or more ANC visits (74% and 46%, respectively), while urban births (61%) were more likely than rural births (26%) to be delivered in a health facility (16). Plausibly, the poor reproductive and maternal health services utilization, especially among the disadvantaged rural women compared to their urban counterparts might be attributed to many factors including household wealth poverty and decision-making autonomy. In Nigeria, some previous studies on the associated factors with the utilization of reproductive and maternal health services (17–19) have not adequately taken into cognizance the disadvantaged rural women. This study becomes relevant in Nigeria which is part of the five countries with the largest populations living in extreme poverty and accounts for about 23% of the world's poor (20). In Nigeria, there is evidence that 84.6% of people in rural areas between 2018 and 2019 lived below the $1.90 poverty line (21).

This study examined household poverty-wealth and decision-making autonomy as predictors of reproductive and maternal health services utilization among rural women in Nigeria. The outcome is expected to provide up-to-date information, relevant policy and programmatic recommendations towards achieving sustainable development goals targets of universal access to quality reproductive and maternal health services; and reducing maternal and child deaths.

## Methods

The study utilized data obtained from the 2018 Nigeria Demographic and Health Survey (NDHS). The survey is a cross-sectional study and data were generated using standardized interviewer-administered questionnaires from a nationally representative sample of women aged (15–49) on socioeconomic, demographic and health variables. For this study, the analyzes covered a weighted sample of 13,151 currently married and cohabiting rural women who were sexually active and reported to have given birth to at least a child in the five years that preceded the survey (i.e. 2013–2018).

The outcome variables selected for this study were based on empirical evidence which includes; 1) the use of contraceptive methods, 2) the number of ANC visits, 3) facility delivery services and 4) the postnatal check provider. Information on these outcome variables as generated from the 2018 NDHS was re-categorized from their original frequency ranges in the dataset. Therefore, women who used a modern contraceptive method, had at least four ANC visits during their most recent pregnancy, delivered in a public or private hospital and skilled postnatal check of a mother during the first 2 days after childbirth/before discharge from a doctor, nurse/midwife or auxiliary nurse/midwife were categorized as ‘1’ and ‘0’ if otherwise.

The main explanatory variables in this study are household poverty-wealth and women's decision-making measures including the following three subjects: 1) decision on respondent's healthcare, 2) decision on large household purchases, and 3) decision on how to spend respondent's earnings. Therefore, women who made independent decisions on any of the three subjects represent decision-making autonomy and may influence seeking healthcare for themselves (22). In this context, household poverty-wealth was estimated as adopted in a previous study to explain household poverty-wealth status in African standards where there is high inequality in income distribution (23). Some covariates influencing the outcome variables including age, education, work status, region, distance to health facility and health insurance were included in the analysis as control variables based on empirical evidence.

Data analysis was conducted with Stata software (version 15) at univariate, bivariate and multivariate levels. The dataset was carefully checked for missing values which were excluded and weighted with the appropriate sampling weights as per the Demographic and Health Survey sampling scheme. At the bivariate level, unadjusted logistic regression as shown in Table 2 was employed to investigate the association between the outcome and explanatory variables. Table 3 presented the adjusted logistic regression at the multivariate level to examine the odds of using reproductive and maternal health services. The statistical significance was set at *p*<0.05 and measures of association were expressed as odds ratio with 95% confidence intervals.

**Table 2:** Unadjusted and bivariate analysis of the influence of household poverty-wealth and decision-making autonomy on reproductive and maternal healthcare services utilization, 2018 Nigeria DHS

Variables/categories	Contraceptive method	Antenatal care visit	Place of delivery	Postnatal care provider
	
	OR(95% CI)	OR(95% CI)	OR(95% CI)	OR (95% CI)
**Household poverty-wealth**				
Rich (Ref.)	1.00	1.00	1.00	1.00
Middle	0.67(0.56–0.77)§	0.45(0.39–0.51)§	0.37(0.33–0.42)§	0.32(0.17–0.62)**
Poor	0.29(0.25–0.34)§	0.17(0.15–0.19)§	0.10(0.09–0.11)§	0.10(0.06–0.18)§
**Decision on respondent's healthcare**				
Husband/partner alone (Ref.)	1.00	1.00	1.00	1.00
Respondent and partner	2.87(2.53–3.26)§	2.51(2.32–2.72)§	3.10(2.85–3.37)§	1.35(94–1.94)
Respondent alone	2.58(2.11–3.16)§	2.10(1.83–2.41)§	2.82(2.45–3.23)§	0.73(0.45–1.17)*
**Decision on large household purchase**				
Husband/partner alone (Ref.)	1.00	1.00	1.00	1.00
Respondent and partner	2.53(2.23–2.86)§	2.21(2.04–2.39)§	2.80(2.58–3.04)§	1.28(0.91–1.80)
Respondent alone	1.98(1.49–2.62)§	2.12(1.76–2.55)§	3.99(3.32–4.79)§	1.27(0.58–2.78)
**Decision on how to spend** **respondent's earnings**				
Husband/partner alone (Ref.)	1.00	1.00	1.00	1.00
Respondent and partner	2.14(1.60–2.85)§	2.67(2.22–3.21)§	2.92(2.42–3.53)§	3.58(1.72–7.42)**
Respondent alone	1.03(0.80–1.35)	1.26(1.09–1.46)**	1.01(0.86–1.19)	3.00(1.66–5.40)§
**Age**				
15 – 24 (Ref.)	1.00	1.00	1.00	1.00
25 – 34	1.63(1.39–1.92)§	1.26(1.16–1.37)§	1.28(1.17–1.40)§	1.58(1.08–2.33)*
35 +	2.04(1.72–2.42)§	1.26(1.15–1.38)§	1.28(1.15–1.41)§	1.41(0.93–2.15)
**Educational attainment**				
No education (Ref.)	1.00	1.00	1.00	1.00
Primary	3.56(2.99–4.23)§	3.15(2.85–3.49)§	4.52(4.04–5.05)§	2.47(1.58–3.85)§
Secondary or higher	5.23(4.54–6.03)§	5.96(5.45–6.52)§	11.87(10.78–13.07)§	4.32(2.99–6.25)§
**Employment status**				
Not working (Ref.)	1.00	1.00	1.00	1.00
currently working	1.91(1.66–2.20)§	1.93(1.80–2.08)§	2.30(2.11–2.50)§	1.40(0.98–2.01)
**Region**				
North-central (Ref.)	1.00	1.00	1.00	1.00
North-east	0.64(0.55–0.76)§	0.66(0.60–0.74)§	0.28(0.25–0.31)§	0.23(0.14–0.35)§
North-west	0.26(0.22–0.32)§	0.53(0.48–0.58)§	0.13(0.12–0.15)§	0.32(0.19–0.51)§
South-east	1.02(0.82–1.30)	7.31(5.76–9.27)§	6.80(5.46–8.47)§	2.77(1.15–6.71)*
South-south	1.20(0.98–1.46)	1.51(1.31–1.74)§	0.87(0.75–1.00)	5.77(1.37–24.31)*
South-west	1.36(1.08–1.71)*	4.35(3.53–5.36)§	4.23(3.47–5.15)§	2.33(1.02–5.35)
**Distance to the health facility**				
Big problem (Ref.)	1.00	1.00	1.00	1.00
Not a big problem	1.44(1.27–1.64)§	2.14(2.00–2.30)§	1.80(1.66–1.95)§	1.83(1.31–2.54)§
**Covered by health insurance**				
No (Ref.)	1.00	1.00	1.00	1.00
Yes	1.59(1.01–2.50)*	2.40(1.72–3.35)§	2.79(2.04–3.81)§	3.74(0.52–27.14)

* *p* < 0.05

** *p* < 0.01

§ p < 0.001

Ref.= reference category

**Table 3:** Adjusted logistic regression analysis of reproductive and maternal healthcare services utilization according to the explanatory variables, 2018 Nigeria DHS

Variables/categories	Contraceptive method	Antenatal care visit	Place of delivery	Postnatal care provider
	
	aOR(95% CI)	aOR(95% CI)	aOR(95% CI)	aOR(95% CI)
**Household poverty-wealth**				
Rich (Ref.)	1.00	1.00	1.00	1.00
Middle	0.93(0.75–1.14)	0.67(0.56–0.80)§	0.63(0.53–0.75)§	0.61(0.25–1.48)
Poor	0.66(0.52–0.84)**	0.43(0.36–0.51)§	0.35(0.29–0.42)§	0.36(0.15–0.88)*
**Decision on respondent's** **healthcare**				
Husband/partner alone and other (Ref.)	1.00	1.00	1.00	1.00
Respondent and partner	1.23(0.94–1.60)	1.20(1.00–1.42)*	1.24(1.02–1.52)*	0.85(0.38–1.89)
Respondent alone	1.51(1.12–2.05)**	1.28(1.03–1.56)*	1.20(0.94–1.54)	0.97(0.31–3.03)
**Decision on large household** **purchase**				
Husband/partner alone and other (Ref.)	1.00	1.00	1.00	1.00
Respondent and partner	1.36(1.06–1.76)*	1.03(0.87–1.22)	0.87(0.72–1.06)	0.67(0.30–1.51)
Respondent alone	1.12(0.76–1.64)	1.12(0.83–1.52)	1.23(0.90–1.69)	0.47(0.13–1.67)
**Decision on how to spend respondent's** **earnings**				
Husband/partner alone and other (Ref.)	1.00	1.00	1.00	1.00
Respondent and partner	1.09(0.79–1.50)	1.35(1.08–1.69)**	1.28(1.00–1.64)	2.74(1.14–6.59)*
Respondent alone	0.95(0.72–1.27)	1.40(1.18–1.66)§	1.25(1.02–1.54)*	4.39(2.24–8.62)§
**Age**				
15 – 24 (Ref.)	1.00	1.00	1.00	1.00
25 – 34	1.47(1.15–1.87)**	1.08(0.94–1.24)	1.01(0.86–1.18)	1.17(0.62–2.22)
35 +	1.88(1.45–2.42)§	1.11(0.95–1.28)	1.08(0.90–1.29)	1.01(0.52–1.97)
**Educational attainment**				
No education (Ref.)	1.00	1.00	1.00	1.00
Primary	2.70(2.09–3.49)§	2.00(1.72–2.33)§	2.30(1.94–2.72)§	2.17(1.10–4.29)*
Secondary or higher	3.78(2.93–4.87)§	2.97(2.52–3.50)§	3.97(3.34–4.71)§	3.38(1.66–6.89)**
**Employment status**				
Not working (Ref.)	1.00	1.00	1.00	-
currently working	1.20(0.77–1.88)	0.76(0.61–0.95)*	0.72(0.55–0.96)*	-
**Region**				
North-central (Ref.)	1.00	1.00	1.00	1.00
North-east	0.56(0.43–0.75)§	1.22(1.02–1.46)*	0.44(0.36–0.53)§	0.63(0.30–1.33)
North-west	0.51(0.39–0.67)§	0.98(0.83–1.17)	0.23(0.19–0.28)§	0.44(0.21–0.91)*
South-east	0.36(0.27–0.50)§	3.28(2.41–4.46)§	2.54(1.90–3.40)§	1.56(0.53–4.59)
South-south	0.49(0.38–0.63)§	0.79(0.64–0.96)*	0.30(0.25–0.38)§	4.87(0.62–38.10)
South-west	0.64(0.48–0.84)**	3.07(2.36–4.00)§	2.46(1.91–3.17)§	2.37(0.87–6.43)
**Distance to the health facility**				
Big problem (Ref.)	1.00	1.00	1.00	1.00
Not a big problem	1.24(1.02–1.50)*	1.92(1.71–2.16)§	1.47(1.26–1.68)§	2.25(1.30–3.90)**
**Covered by health insurance**				
No (Ref.)	1.00	1.00	1.00	-
Yes	0.93(0.53–1.63)	1.21(0.71–2.05)	1.71(0.96–3.02)	-

* *p* < 0.05

** *p* < 0.01

§ *p* < 0.001

Ref.= reference category

## Results

**Description of the study population**: The weighted descriptive statistics of the women as presented in Table 1 showed that 45.7% were aged 25–34 years; over one-half had no formal education and were employed. The largest proportion (35.5%) resided in the North-west region, 63.5% reported that distance to health facilities was not a big problem, while 98.8% were not covered by health insurance. The results further showed that 63.7% of the women were found in poor households. Over two-thirds of the women lacked decision-making autonomy on their healthcare and large household purchases, while 71.9% made independent decisions on how to spend their earnings.

**Table 1:** Distribution of the characteristics of the study population, 2018 Nigeria DHS (N = 13,151)

Variables	Number (%)
**Age *(Mean)***	29.3 years
15 – 24	3,574(27.2)
25 – 34	6,017(45.7)
35 +	3,560(27.1)
**Educational attainment**	
No education	7,667(58.3)
Primary	1,998(15.2)
Secondary or higher	3,486(26.5)
**Employment status**	
Not working	4,622(35.2)
currently working	8,529(64.9)
**Region**	
North-central	2,504(19.0)
North-east	3,391(25.8)
North-west	4,672(35.5)
South-east	738(5.6)
South-south	1,151(8.8)
South-west	695(5.3)
**Distance to the health facility**	
Big problem	4,801(36.5)
Not a big problem	8,350(63.5)
**Covered by health insurance**	
No	12,991(98.8)
Yes	160(1.2)
**Household poverty-wealth**	
Rich	2,114(16.1)
Middle	2,655(20.2)
Poor	8,382(63.7)
**Decision on respondent's** **healthcare**	
Husband/partner alone	8,742(66.5)
Respondent and partner	3,471(26.4)
Respondent alone	938(7.1)
**Decision on large household** **purchase**	
Husband/partner alone	9,061(68.9)
Respondent and partner	3,594(27.3)
Respondent alone	496(3.8)
**Decision on how to spend** **respondent's earnings**	
Husband/partner alone	814(11.5)
Respondent and partner	1,177(16.6)
Respondent alone	5,083(71.9)

**Figure 1:**
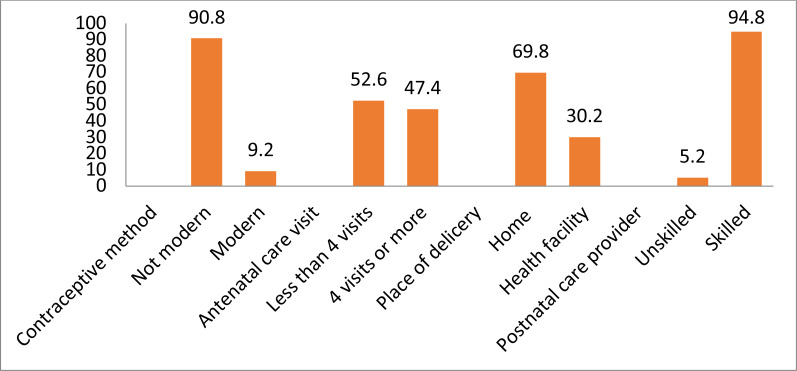
Utilisation of reproductive and maternal healthcare services (%).

**Reproductive and maternal healthcare services utilization**: Figure 1 shows that 90.8% of rural women reported not having used modern contraceptive methods, 53% had less than 4 ANC visits, 69.8% delivered at home and 94.8% received postnatal check-ups from skilled health providers during the first 2 days after delivery.

**Unadjusted and bivariate analysis**: The unadjusted and bivariate results as presented in Table 2 showed that all the main explanatory variables and covariates are significantly associated with the use of modern contraceptive methods, having at least four ANC visits and delivery in the health facility at *p*<0.05. However, except for employment status and health insurance coverage, all the main explanatory variables and other covariates are significantly associated with receiving postnatal care from a skilled provider during the first 2 days after childbirth at *p*<0.05.

**Adjusted multivariate analysis of the utilization of reproductive and maternal healthcare services by explanatory variables**: The adjusted logistic regression analysis results presented in Table 3 showed that rural women from poor households compared to those from rich households were less likely to use modern contraceptive methods (aOR: 0.66, 95% CI: 0.52–0.84), have at least four ANC visits (aOR: 0.43, 95% CI: 0.36–0.51), deliver in a health facility (aOR: 0.35, 95% CI: 0.29–0.42) and receive postnatal care from a skilled provider (aOR: 0.36, 95% CI: 0.15–0.88). Additionally, rural women who enjoyed decision-making autonomy regarding their healthcare were significantly more likely to use modern contraceptive methods (aOR: 1.51, 95% CI: 1.12–2.05) and have at least four ANC visits during pregnancy (aOR: 1.28, 95% CI: 1.03–1.56). Also, rural women's decision-making autonomy on how to spend their earnings significantly increased the likelihood of using maternal healthcare services. Concerning the covariates, an increase in the age of rural women increased the likelihood of using modern contraceptive methods, while those who had higher educational attainment and reported that distance to health facilities was not a big problem were significantly more likely to use reproductive and maternal healthcare services. Surprisingly, rural women who were currently employed were significantly less likely to have at least four ANC visits during pregnancy and deliver in a health facility. There were significant regional variations regarding the utilization of reproductive and maternal healthcare services.

## Discussion

This study examined the influence of household poverty-wealth and decision-making autonomy on reproductive and maternal health services among rural women in Nigeria. The findings of this study revealed that despite the efforts in encouraging the use of modern contraceptive methods, the prevalence of modern contraceptives among rural women is still alarmingly low corroborating different studies in Congo (24) and Ethiopia (25). This finding could further reflect the wide rural-urban differences in the level of awareness and utilization of modern contraceptive methods, as well as reveal the high risk of pregnancy among rural women, especially during the postpartum period in Nigeria. Optimal ANC visits and delivery in a health facility were poorly utilized among rural women. The result on delivery in a health facility corroborates a study in Ethiopia which showed that urban women are more likely to utilize safe delivery services than rural women (26). Also, in line with a previous study that most rural women who delivered at home further sought skilled medical attention (27), this study revealed that some rural women, who delivered at home, subsequently received PHC from skilled providers during the first 2 days after childbirth. This is an indication that most rural women follow skilled postnatal care irrespective of the place of delivery. The finding is in contrast with previous observations in Malawi (28) and Ethiopia (29) that women who were from rural areas were less likely to receive skilled postnatal care compared with their counterparts from urban settings.

The findings further revealed that rural women found in poor households were less likely to use modern contraceptive methods relative to those in rich households. This corroborates previous studies in sub-Saharan Africa including Nigeria that the poverty-wealth dimension in rural areas is a big factor contributing to the lack of modern contraceptive use (30–31). Also, the findings showed that rural women from poor households compared to those found in rich households were significantly less likely to have at least 4 ANC visits, deliver in a health facility and receive PHC from a skilled provider. The findings validate the observations in previous studies on the influence of low-wealth households on adequate maternal healthcare services utilization in Ethiopia and Nigeria (32–33). This could be attributed to the fact that the chances of using reproductive and maternal health services increase as rural women do not have financial limitations (34).

This study showed that rural women's decision-making autonomy regarding their healthcare is a significant predictor of using modern contraceptive methods and having at least four ANC visits during pregnancy. The findings corroborate previous studies that women who can make independent decisions on their reproductive health were more likely than those whose husbands solely made such decisions to use modern contraceptives (24, 35) and maternal healthcare services in SSA (18). This plausibly explains that most typical women who still rely on the husband for key decision making including healthcare may not enjoy spousal support for utilizing contraception (31). Also, corroborating a previous study of 31 sub-Saharan African countries (36), the findings of this study further showed that rural women's autonomy on how to spend their earnings was strongly associated with the use of maternal healthcare services. Consequently, the poor utilization of reproductive and maternal health services among rural women could be attributed to a lack of financial resources, which leaves them to the decisions of their husbands (37).

The findings of this study further confirmed an observation that younger women were less likely to use modern contraceptives compared to their older counterparts (38). Plausibly, older women may adopt the use of modern contraceptives to avoid unwanted pregnancies and limit or space childbirths relative to younger women with few or no children. The findings further showed that rural women with formal education were significantly more likely to use modern contraceptives and quality maternal health services. This confirms previous observations in Nigeria (35) and Kenya (39), that education empowers women with better knowledge of the availability and benefits of reproductive and maternal health services. In contrast with a study in Benin (40), this study showed that the likelihood of having at least four ANC visits and delivering in a health facility was reduced among rural women who reported being employed. Consistent with previous studies in SSA (41–42), this study showed that distance to health a facility not being a big problem was a protective factor in using reproductive and maternal health services among rural women. There were variations regarding the utilization of reproductive and maternal health services among rural women as previously observed in a study in Nigeria (43–44).

In conclusion, this study found that the prevalence of modern contraceptives is still alarmingly low, while optimal ANC visits during pregnancy and delivery in health facilities are poorly utilized among rural women. Some rural women who delivered at home subsequently sought skilled medical attention from health facilities. Generally, reproductive and maternal health services utilization were negatively influenced by household poverty-wealth. Women's decision-making autonomy regarding their healthcare and earnings are significant predictors of reproductive and maternal health services utilization. There is a need for some unified skilled postnatal home-visit services outside health facilities through linkages with healthcare providers. Also, the government should formulate more pragmatic policies that will create awareness, educate and promote adequate reproductive and maternal healthcare services utilization among disadvantaged women.

The study has some limitations including the use of secondary data from a cross-sectional study which restricts study variables. As a result, the cause-effect association between the explanatory and outcome variables is rather temporary and may not be ascertained. However, the findings are essential for the formulation of more pragmatic policies geared toward achieving universal access to reproductive and maternal healthcare services.
